# Anthelmintic resistance and homeostatic plasticity (*Brugia malayi*)

**DOI:** 10.1038/s41598-021-93911-4

**Published:** 2021-07-14

**Authors:** Sudhanva S. Kashyap, Saurabh Verma, Mark McHugh, Mengisteab Wolday, Paul D. Williams, Alan P. Robertson, Richard J. Martin

**Affiliations:** Department of Biomedical Sciences, Iowa State University, Ames, IA 50011 USA

**Keywords:** Biological techniques, Cell biology, Drug discovery, Molecular biology, Physiology, Diseases, Medical research

## Abstract

Homeostatic plasticity refers to the capacity of excitable cells to regulate their activity to make compensatory adjustments to long-lasting stimulation. It is found across the spectrum of vertebrate and invertebrate species and is driven by changes in cytosolic calcium; it has not been explored in parasitic nematodes when treated with therapeutic drugs. Here we have studied the adaptation of *Brugia malayi* to exposure to the anthelmintic, levamisole that activates muscle AChR ion-channels. We found three phases of the *Brugia malayi* motility responses as they adapted to levamisole: an initial spastic paralysis; a flaccid paralysis that follows; and finally, a recovery of motility with loss of sensitivity to levamisole at 4 h. Motility, calcium-imaging, patch-clamp and molecular experiments showed the muscle AChRs are dynamic with mechanisms that adjust their subtype composition and sensitivity to levamisole. This homeostatic plasticity allows the parasite to adapt resisting the anthelmintic.

## Introduction

Nematode parasites are more complex than bacteria and viruses. They have a 42–700 mega-base genome^1^, a nervous system^2^ that allow learning^3,4^ and different motor responses to chemical stimulation^5^. We expect that they have more complex mechanisms than viruses and bacteria to adapt and resist exposure to anthelmintic drugs.

How do nematode parasites accommodate and adapt to anthelmintic drugs designed to eliminate them? Conventional understanding of anthelmintic resistance is that it is genetically based on: (i) mutation and/or selective elimination of sensitive anthelmintic targets; (ii) increased drug metabolism and; (iii) reduced entry and/or excretion of the anthelmintic^6,7^. What are additional intrinsic mechanisms that allow individuals to become resistant to anthelmintics?

Homeostatic plasticity^8,9^ refers to the capacity of excitable cells to regulate their activity to make compensatory adjustments to long-lasting stimulation. The plasticity involves changes in ion-channels in nerves and muscles of vertebrate and invertebrate species including nematodes^10–12^ and is triggered by changes in cytosolic calcium^13,14^. It has not been explored in parasitic nematodes during applications of anthelmintic drugs. The speed and level of habituation and adaptation to an anthelmintic will bear on their ability to survive and resist treatment.

We study the filarial nematode parasite, *Brugia malayi*^15–17^*.* Filariae are transmitted by biting insects that feed on infected hosts’ blood and pass the parasite onto a subsequent uninfected host. A group of filarial nematodes like *B. malayi* that locate as adults in the host lymphatic system produces the condition known as lymphatic filariasis. They block drainage of the lymphatics, inducing gross swelling of the limbs, itching and skin infections that produce the clinical condition, elephantiasis. There are an estimated 70 million people infected with lymphatic filariasis^18^ in 52 countries. There are no effective vaccines, so Mass Drug Administration (MDA) to control and prevent infection is the only practical option. The use of MDA gives rise to concerns about resistance and an interest in the mechanisms that the parasites use to overcome the effects of anthelmintics.

*Brugia malayi* are a tractable nematode parasite for study. They are available from an NIH-supported facility, FR3; they are amenable to knockdown; their genome is available; RNAi is a tractable procedure; their motility can be followed with Worminator and; patch-clamp recording of current responses to anthelmintic drugs can be made. In preliminary experiments we have observed that exposure of *B. malayi* to the anthelmintic levamisole*,* produces paralysis which wanes over time allowing the parasite to recover motility.

Here we report quantitative motility studies, calcium fluorescence observations, patch-clamp studies and molecular observations seeking mechanistic explanations for the levamisole habituation. We followed currents in muscle cells induced by levamisole and observed that levamisole activated receptors (AChRs) over a period of 20–30 min: we observed a selective loss of levamisole activated currents but not acetylcholine activated currents. On the naïve muscle cell there are at least four separable types of AChRs. These are modified during maintained application of levamisole and are affected by knockdown of *nra-2 *^19,20^ that is involved in selection of protein complexes that exit the endoplasmic reticulum. We observed increases in the level of *unc-38* message with a reduction in *nra-2* message that drive a change in the AChR subtypes present on muscle. We conclude that the AChRs of the parasite habituate and suggest that the composition of the AChR subunits is plastic allowing the parasite to habituate to the presence the anthelmintic, levamisole. Such plasticity will support survival of nematode parasites during treatment and facilitate resistance to the anthelmintic.

## Methods

### Parasite maintenance

*Brugia malayi* adult worms were obtained from the NIH/NIAID Filariasis Research Reagent Resource Center (FR3; College of Veterinary Medicine, University of Georgia, Athens, GA, USA). Adult worms were maintained in Roswell Park Memorial Institute (RPMI) 1640 media (Life Technologies, USA) supplemented with 10% heat-inactivated fetal bovine serum (FBS, Fisher Scientific, USA) and 1% Antibiotic–Antimycotic (Life Technologies, USA). The worms were stored individually in 24 well culture plates containing 1 ml of supplemented RPMI-1640 media and placed in an incubator at 37 °C supplemented with 5% CO_2_ as previously explained^15–17^.

### Drugs

Levamisole, acetylcholine, pyrantel, morantel, and nicotine were obtained from Sigma Aldrich (St. Louis, MO, USA) and stock concentrations of levamisole HCl (100 mM), pyrantel citrate (30 mM), morantel citrate (30 mM), nicotine (30 mM), and acetylcholine chloride (100 mM) were prepared in distilled water and diluted in bath solution to obtain final concentration. Acetylcholine chloride was made up freshly for each days’ experiment. For motility assays, the recording solution was RPMI-1640, while for whole-cell recordings drugs were diluted in recording solution as mentioned below to obtain a final concentration.

### Dissection

*Brugia malayi* body muscle flaps were made using methods modified from *C. elegans*^21,22^. 5 mm lengths of the worm were cut from the anterior region of the *B. malayi* and placed in the bath solution (composition below). The base of the chamber was a cover slip coated with a layer of thin Sylgard^R^. The length of the worm was fixed down by gluing along one of its sides with Glushield^R^ cyanoacrylate glue (Glustich Inc., Canada). The length of worm was then cut open longitudinally using a tungsten needle. The resulting body muscle flap was then opened, and the cut edge glued down after the reproductive and intestine tissue of the *B. malayi* were removed with fine forceps. The preparation was then viewed under 400× DIC optics with an inverted TE2000-U Nikon microscope. After dissection of the adult worms, recordings were performed at room temperature^15–17,23^. The bath solution was: 23 mM NaCl, 110 mM Na acetate, 5 mM KCl, 6 mM CaCl_2_, 4 mM MgCl_2_, 5 mM HEPES, 10 mM d-glucose, and 11 mM sucrose, pH adjusted to 7.2 with NaOH, ~ 320 mOsmol.

### Whole-cell recording

Muscle flaps were incubated in 1 mg/mL collagenase (Type 1A) in bath solution for 15-120 s and washed 10 times prior to recording. The patch-clamp technique explained below was used to record whole-cell currents from the muscle flaps as explained in Ref.^23^. Patch pipettes were pulled from capillary glass (G85150T; Warner Instruments Inc., Hamden, CT, USA), fire polished and then filled with pipette solution (120 mM KCl, 20 mM KOH, 4 mM MgCl_2_, 5 mM TRIS, 0.25 mM CaCl_2_, 4 mM NaATP, 5 mM EGTA and 36 mM sucrose (pH 7.2 with KOH), ~ 315–330 mOsmol). Pipettes with resistances of 3–5 MΩ were used. A 1 cm region near the tip of the electrode was covered with Sylgard™ to reduce background noise and improve frequency responses. Giga ohm seal was formed before breaking the membrane with suction. The preparation was continuously perfused in bath solution at 2 mL/min. The current signal was amplified by an Axopatch 200B amplifier (Molecular Devices, CA, USA) filtered at 2 kHz (three-pole Bessel filter), and sampled at 25 kHz, digitized with a Digidata 1440A (Molecular Devices, CA, USA).

### Calcium imaging and Fluo-3 injections

Patch pipettes were filled with the pipette solution described above with added 5 µM Fluo-3 penta-ammonium diluted in DMSO added before each experiment and were kept in a dark environment to prevent degradation of the dye. Pipettes with resistances of 1.8–3 MΩ were used. After breaking into cells, they were left for a minimum of 10 min to allow Fluo-3 to diffuse into the muscle cell in the presence of bath solution containing 1 mM CaCl_2_^24^. At the start of recordings, cells were consistently perfused with bath solution containing: 23 mM NaCl, 110 mM Na acetate, 5 mM KCl, 1 mM CaCl_2_, 4 mM MgCl_2_, 5 mM HEPES, 10 mM d-glucose, and 11 mM sucrose, pH adjusted to 7.2 with NaOH, ~ 320 mOsmol at a rate of 1.5 mL/min. Long term levamisole responses were achieved by continuously exposing cells to 30 µM levamisole diluted in bath solution described above for 40–60 min. Responses in muscles exposed to 10 mM CaCl_2_ were used as positive controls.

All recordings were performed using a Nikon Eclipse TE3000 microscope (20×/0.45 Nikon PlanFluor objective), fitted with a Photometrics Retiga R1 Camera (Photometrics, Surrey, BC, Canada)^24,25^. Light control was achieved using a Lambda 10-2 two-filter wheel system with a shutter controller (Lambda Instruments, Switzerland). Filter wheel one was set on a green filter (525–530 mm) between the microscope and camera. Filter wheel two was set on the blue filter (490 mm) between a Lambda LS Xenon bulb light box, which delivered light via a fiber optic cable to the microscope (Lambda Instruments, Switzerland), to activate Fluo-3. Blue light emission was controlled using a shutter. Minimal exposure to blue light during recording set up was used to prevent reduction in signal strength.

Calcium signal recordings were acquired and analyzed using MetaFluor 7.10.2 (MDS Analytical Technologies, Sunnyvale, CA, USA) with exposure settings at 250 ms with 2 × binning. Maximal Ca^2+^ signal amplitudes (ΔF) were calculated using the equation F1 − F0/F0 × 100, where F1 is the fluorescent value and F0 is the baseline value. All F0 values were determined as being the value at the time the stimulus was applied to the sample for all recordings analyzed^24,25^.

### RNA extraction and cDNA synthesis

RNA extraction and cDNA synthesis was performed as previously described^15–17,23^. *B. malayi* adult worms were snap frozen and crushed into fine powder in a 1.5 mL micro-centrifuge tube using Kimble™ Kontes™ Pellet Pestle™ (Fisher Scientific, USA). Total RNA was extracted using TRIzol^®^ Reagent (Life Technologies, USA) according to the manufacturer’s instructions. About 1 µg of total RNA was used to synthesize cDNA using SuperScript^®^ VILO™ Master Mix (Life Technologies, USA). Samples were either used to amplify DNA using PCR or stored at − 20 °C for later use.

### Synthesis and delivery of dsRNA

dsRNA was synthesized as explained in^17,26^. Target PCR products were amplified using the primers in Table 1. T7 promoter sequence 5′-TAATACGACTCACTATAG-3′ was used as an overhang to produce T7 labelled PCR products for dsRNA synthesis. dsRNA was synthesized using the T7 RiboMAX™ Express RNAi kit (Promega, USA) according to the manufacturer’s instructions. Adult *B. malayi* were soaked in RPMI media containing 30–60 µg/mL of target and control dsRNA for four days. dsRNA for *lacZ* was used as off-target control. Motility experiments on RNAi worms were performed after 4 days. Worms that were not used in the motility assay were cut into two pieces—one for electrophysiology recordings and the other was snap frozen in liquid nitrogen and stored at − 80 °C for transcript analysis by qPCR.

**Table 1:** List of primers used in this study.

Primer name	Description	Sequence 5′–3′
*nra-2f*	*Bma nra-2 dsRNA 5’*	GCAGCAATTGTTAAATGCAA
*nra-2r*	*Bma nra-2 dsRNA 3’*	ATCCTGGTATTGCTGAATGG
*unc-38f*	*Bma unc-38 dsRNA 5’*	TACTATCCGTCCGTCGAGTG
*unc-38r*	*Bma unc-38 dsRNA 3’*	TTCACCACTATGCGATGGTA
*gapdhf*	*Bma gapdh dsRNA* 5′	GACGCTTCAAGGGAAGTGTTTCTG
*gapdhr*	*Bma gapdh* *dsRNA* 3′	GTTTTGGCCAGCACCACGAC
LacZf	LacZ dsRNA 5′	CGTAATCATGGTCATAGCTGTTTC
LacZr	LacZ dsRNA 3′	CTTTTGCTGGCCTTTTGCTC
*acr-16f*	*Bma acr-16 dsRNA 5′*	CGACCAGGAGTTCATCTCTC
*acr-16r*	*Bma acr-16 dsRNA 3′*	GAAATTGGGCTCTTTCCATT
*acr-26f*	*Bma acr-26 dsRNA 5′*	CTCAATTAAATTCGGCTCGT
*acr-26r*	*Bma acr-26 dsRNA 5′*	AGCGTCTTCCGTCTGATATG
*unc-63f*	*Bma unc-63 dsRNA 5′*	CAGAAACATTGCTTGGCTTT
*unc-63r*	*Bma unc-63 dsRNA 3′*	AGGTGATTCACAGCATGGAT
*unc-29f*	*Bma unc-29 dsRNA 5′*	CCTCATCCACAATCCCACTA
*unc-29r*	*Bma unc-29 dsRNA 3′*	CGGTTTTGGTCTTTGCATAC
*acr-8f*	*Bma acr-8 dsRNA 5′*	CGGTTTCCAAATTGATGTTC
*acr-8r*	*Bma acr-8 dsRNA 3′*	AGGATACAGGCGTTCATGTC

### Analysis of transcript levels

cDNA from dsRNA treated worms were amplified using target and reference gene (*Bma gapdh*) primers (Table 1). These genes were amplified in triplicate by quantitative real-time PCR (qPCR) using the QuantStudio™ 3–96 well 0.1 mL Block Real time PCR detection system (Thermofisher) and PowerUp™ SYBR® Green Supermix (Thermofisher, USA). Cycling conditions used: 95 °C × 10 min, 40 × (95 °C × 10 s, 55 °C × 30 s). PCR efficiencies were calculated using the Design and Analysis Suite (Thermofisher, USA). Relative quantification of target gene knockdown was estimated by the ∆∆Ct method^27^.

### *B. malayi* in vitro motility studies

Motility assays were carried out on the Worminator system in a 24-well tissue culture plate (1 worm/well) containing 1 mL of RPMI-media with l-glutamine as described (Marcelino et al. 2012). This assay assesses motility through pixel displacement of each worm over time, giving an output of Mean Movement Units (MMUs). An active compound that inhibits motility reduces the MMUs.

To determine the potency of levamisole on adult female *B. malayi*, worms were treated with various concentrations of levamisole (10 nM, 30 nM, 100 nM, 300 nM, 1 µM, 3 µM, 10 µM, 30 µM and 100 µM). Worm motility was then recorded prior to the addition of levamisole and 30 s post treatment for each drug concentration. From this we were able generate a concentration–response curve at 30 s and determine IC_50_ values of levamisole. Percent motility was also calculated as a percentage ratio of motility of worms after treatment at the 30 s time point over motility of naïve worms.

Next, to study the long-term effects of various concentrations of levamisole on adult female *B. malayi* motility, we conducted motility assays over a 4-h period. Worm motility was recorded prior to the addition of levamisole, 16 s following the addition of levamisole and at 10, 20, 30, 40, 50, 60, 90, 120, 150, 180 and 240-min post treatment to generate a concentration- and time-course response analysis.

To quantify the effect of long-term application of levamisole at high concentrations, adult female *B. malayi* were assigned into control or drug treatment (n = 4/batch) groups (1 worm/well). Worm motility was recorded prior to the addition of levamisole, immediately following the addition of levamisole and at 30, 60, 90, 120, 150, 180, 210 and 240-min post treatment. Control worms were treated with deionized water, while drug treatment worms were exposed to 100 µM levamisole. Motility was recorded for 30 s for all time points. Three independent experiments were carried out for this study.

To investigate the effects of pyrantel, morantel and nicotine individually on adult female *B. malayi* motility, worms were exposed to each drug individually at a concentration of 10 µM. Control worms were exposed to de-ionized water. Worm motility was recorded as previously described above. Two independent experiments were carried out for this study.

To examine the effects of a second application of levamisole on worms that recovered during the levamisole 4 h treatment, we transferred the worms to fresh RPMI media and added 100 µM after 1 minute or after 120 minutes. As stated previously, worm motility was recorded using the Worminator system prior to the second addition of levamisole, and following addition of the drug. Control worms were treated with de-ionized water.

### Data analysis

The statistical analysis was performed as previously described in (Kashyap et al. 2019; Verma et al. 2020). The data for whole-cell recordings were analyzed with Clampfit 11.1 (Molecular Devices, CA, USA) and GraphPad Prism 5.0 software (Graphpad Software, Inc., La Jolla, CA, USA). The peak current responses from whole-cell recordings were used for analysis. For adult *B. malayi* concentration–response relationships, mean motility was plotted against log concentration. Drug concentrations were log_10_ transformed before analysis. The concentration response curves were fitted using the log inhibitor vs. response equation (variable slope) and the IC_50_ values were calculated from the resulting curves. The responses were plotted as the mean ± SE. Statistical analyses were performed on groups of values by using Two-way ANOVA and Bonferroni post-hoc tests were used to test significance. Paired Student’s *t-*tests were used determine whether there were significant differences between groups where appropriate; two-tailed unpaired Student’s *t*-tests were used when comparing means from different preparations. GraphPad Prism 5 software was used for statistical calculations.

## Results

### Levamisole produces spastic paralysis, then a flaccid paralysis followed by recovery of motility

**Figure 1:**
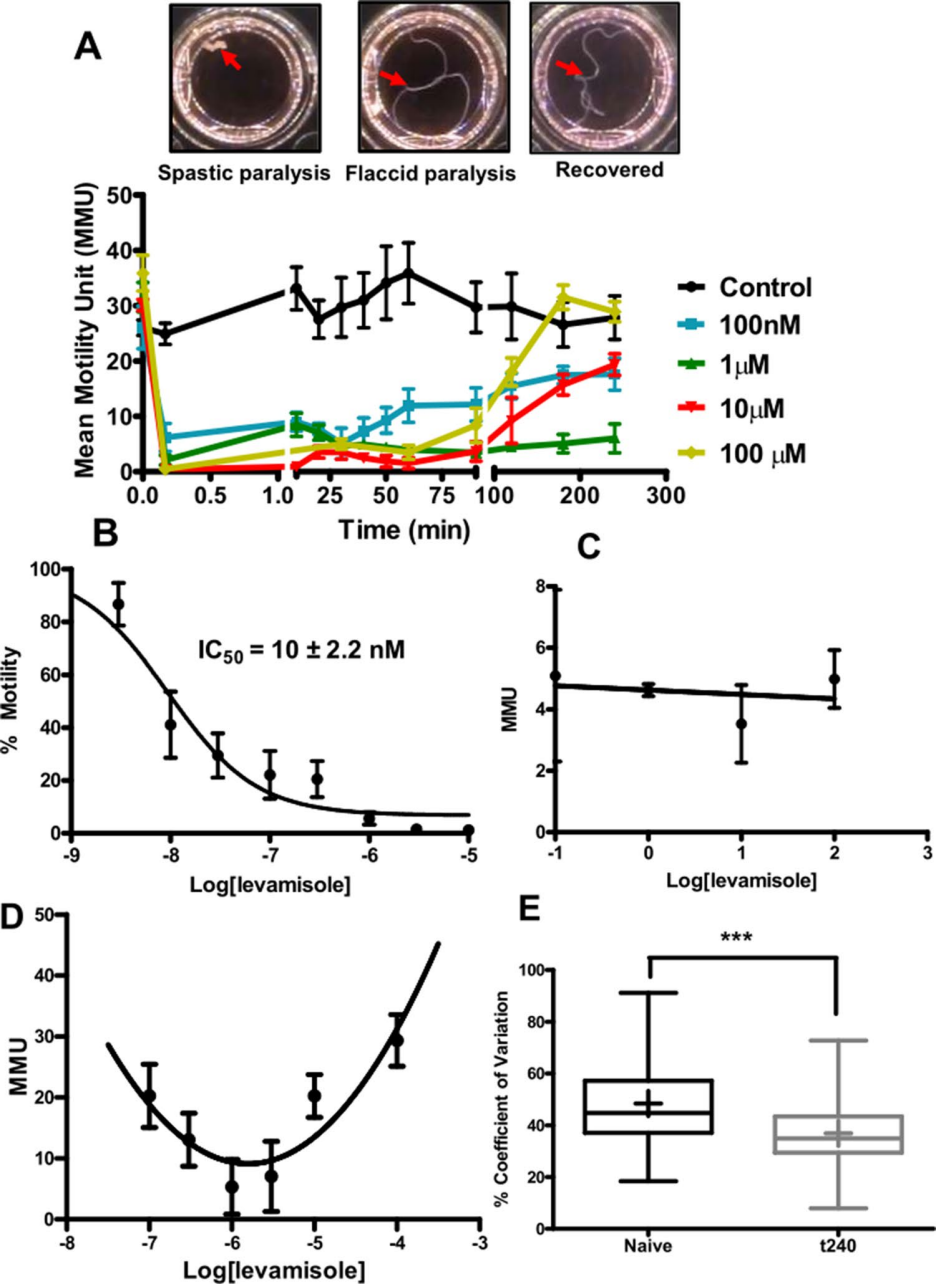
Levamisole is highly potent in paralyzing adult *B. malayi* females, but the worms undergo desensitization within 4 h. (**A**) Images of the time-dependent stages: (i) spastic paralysis as the worms contracted into a ball within 1 min after application of levamisole; (ii) flaccid paralysis as the worms gradually relaxed over the next 15–30 min but did not move and; (iii) the habituation and recovery phase as motility returned over the next 120–180 min. (**B**) Concentration–response analysis of adult *B. malayi* females treated with various concentrations of levamisole. Motility was measured 30 s post levamisole treatment. IC_50_ = 10 ± 2.2 nM; n = 8 per concentration over two biological replicate studies. (**C**) Concentration–response analysis of *B. malayi* adult females treated with various concentrations of levamisole. Mean motility unit (MMU) values were taken at the 30 min time point post levamisole treatment; n = 12 worms per concentration over three biological replicate studies. (**D**) Non-linear concentration-MMU-response analysis of *B. malayi* adult females treated with various concentrations of levamisole T 240-min time point post levamisole treatment; n = 12 worms per concentration over three biological replicate studies. A 2nd order binomial was fitted (r^2^, 0.64) to illustrate the U-shaped relationship was better than a linear fit (low r^2^, 0.24). (**E**) Percent co-efficient of variation in naïve and recovered worms at t240; n = 60 over 15 biological replicate studies. Student’s *t-test* was performed for statistical analysis and p < 0.001 was statistically significant as indicated by ***.

In the following investigations, we explore how *B. malayi* adapt and respond to long term applications of the cholinergic anthelmintic levamisole. Figure 1A shows photographs of 100 µM levamisole treated adult female *B. malayi* at different stages and motility plots for the *B. malayi* following application of different concentrations of levamisole. There were three motility phases: (i) an initial spastic paralysis as the worms contracted into a ball within 1 min after application of levamisole; (ii) a flaccid paralysis as the worms gradually relaxed over the next 15–30 min but did not move and; (iii) the habituation and recovery phase as motility returned over the next 120–180 min. These observations demonstrate that the nematode parasites adapt to exposure to an anthelmintic and that the habituation is time dependent.

Figure 1B shows the concentration-dependent inhibitory effects of levamisole on worm motility during the spastic paralysis measured at 30 s. The *IC*_*50*_ of levamisole was 10 ± 2.2 nM, which highlights the potency of the effects of levamisole. Figure 1C shows the concentration-independent motility response at 30 min during the flaccid paralysis phase, which showed no significant correlation between motility and concentration of levamisole. Inspection of the worms revealed that their bodies were spread out in their respective wells but not moving at this stage.

We followed the motility during the recovery phase over 240 min, where we observed that worms treated with either the highest or the lowest concentrations of levamisole recovered more rapidly, Fig. 1D. The concentration motility response at 240 min was U-shaped with two components: the decreasing motility with levamisole concentration explained by the spastic and flaccid paralysis associated with the opening of more AChRs with increased concentrations and; the recovery of motility that required further study. Although there was nearly full recovery when plotted in mean motility units (MMU: Worminator) with 100 µM at 4 h, there was a detectable difference between the control and recovered pattern of the motility: the recovered worm motility was somewhat more ‘Jerky’ suggesting changes in motor control. The coefficient of variation of the motility of naïve untreated was significantly different to that of the recovered worms, Fig. 1E. We show later that following the recovery, the AChR channel currents are modified.

### Calcium fluorescence and effect of levamisole

**Figure 2:**
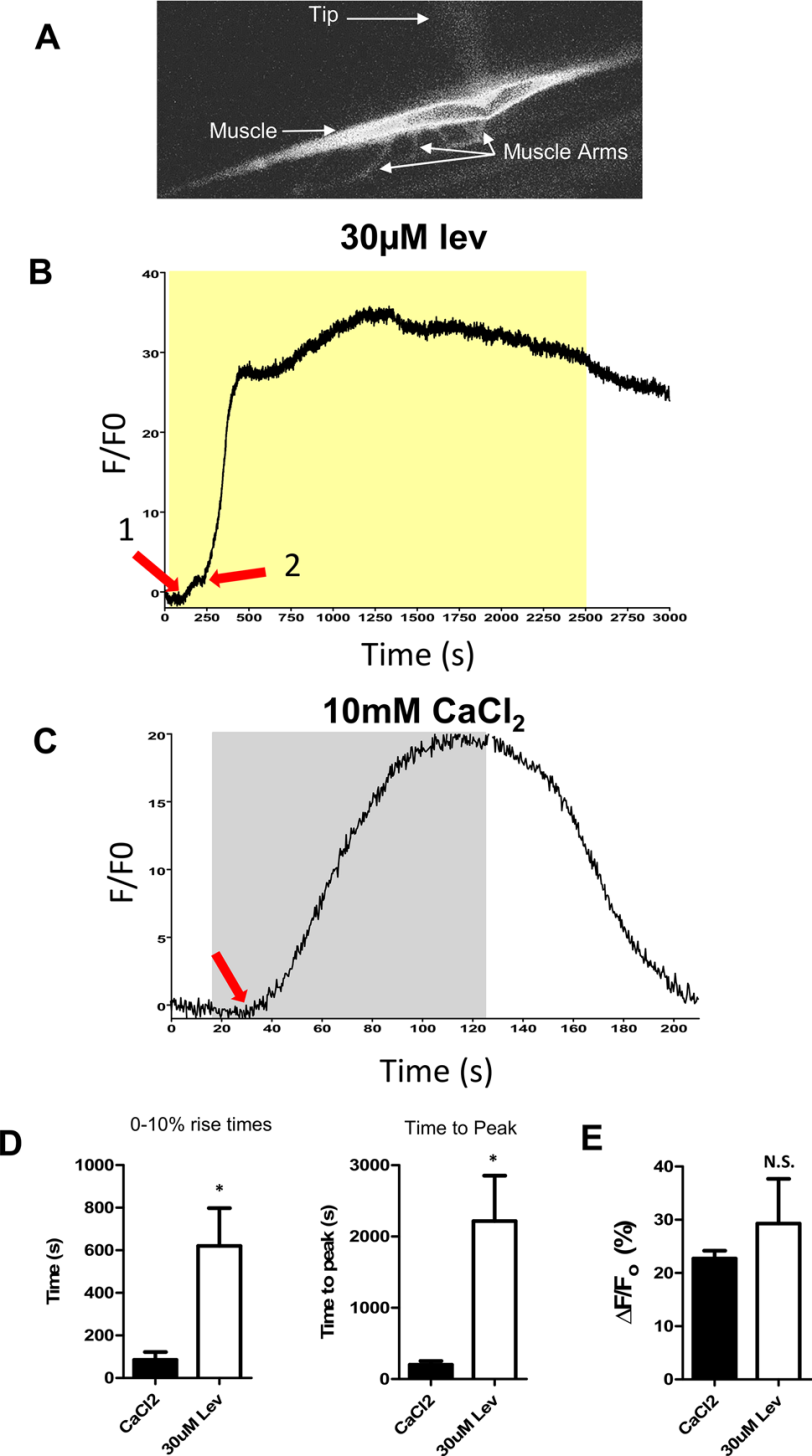
Long-term levamisole application generates a calcium response in *Brugia malayi* muscles injected with Fluo-3. (**A**) Picture of muscle cell taken illustrating fluorescence after injection of 5 µM Fluo-3. The patch-pipetted is just visible (Tip). The muscle arms are shown as is the strap-like muscle. (**B**) Representative trace of a calcium signal in response to long-term application of 30 µM Levamisole. Arrow 1 represents the first initial increase in the calcium signal. Arrow 2 indicates the second, much larger increase. Yellow box indicates levamisole application. (**C**) Representative response to 10 mM CaCl_2_. Arrow represents the initiation of the response. Grey box represents stimulus application. (**D**) Quantification of the average time taken for the calcium signal to reach 10% of the overall amplitude in response to 10 mM CaCl_2_ (black bar) and 30 µM levamisole (White bar). *Significantly different to CaCl_2_ (CaCl_2_ vs lev *p* ≤ 0.0181, *t* = 2.961, *df* = 8, unpaired t-test). Quantification of the average time for the calcium signal to reach peak (100%) in response to 10 mM CaCl_2_ (Black bar) and 30 µM levamisole (White bar). *Significantly different to CaCl_2_ (CaCl_2_ vs Lev *p* ≤ 0.0134, *t* = 3.160, *df* = 8, unpaired t-test. (**E**) Amplitudes of Ca^2+^ signals in the muscle in response to 10 mM CaCl_2_ (Black bar) and 30 µM levamisole (White bar). N.S. not significantly different to CaCl_2_ (CaCl_2_ vs Lev *p* = 0.4622, *t* = 0.7723, *df* = 8, unpaired t-test). All values represented as mean ± SEM. CaCl_2_ recordings *n* = 5 muscles from 5 individual worms. Levamisole recordings *n* = 5 muscles from 5 individual worms.

An increase in cytoplasmic calcium is predicted to follow opening of the calcium permeable ***L***-AChRs^28^ that is followed by a further increase due to intracellular release from ryanodine receptors^29,30^. The effect of maintained applications of levamisole on cytosolic calcium levels was of interest because of calcium’s role in excitation–contraction coupling. Was the flaccid paralysis associated a fall in cytoplasmic calcium? To address this question, we used patch-pipettes to inject Fluo-3 into the muscle cells as a calcium-sensitive indicator, Fig. 2A.

Figure 2B,C shows representative traces of the effect of 30 µM levamisole and 10 mM CaCl_2_ on the Fluo-3 calcium signal. Note that levamisole produces an initial small rise in calcium fluorescence (Fig. 2B arrow: 1) that is followed by a rapid and bigger rise that starts near 250 s (Fig. 2B arrow: 2). There is then a rapid rise that reaches a peak at 22 min (1360 s) before declining slowly, even in the continued presence of levamisole. This peak is during the stage of flaccid paralysis so that a reduced cytosolic calcium does not explain this paralysis. Figure 2C shows the response to the high 10 mM CaCl_2_ bath solution which starts 15 s after the addition of calcium; the calcium signal rises and then is maintained until the bath is perfused with the lower calcium solution when it falls rapidly. Figure 2D shows histograms of the 0–10% rise times and the time to peak (100%) of 30 µM levamisole and 10 mM CaCl_2_ signals, highlighting the slower initiation and peak of the levamisole signal compared to the rapid CaCl_2_ signal. There was no significant difference in the amplitude of the calcium signals between the effect of levamisole and CaCl_2_ (Fig. 2E). The delay, the biphasic nature of the response to levamisole in contrast to the application of high calcium bathing solutions suggest that the initial rise in cytosolic calcium by levamisole is mediated by the plasma membrane AChRs and the secondary larger delayed rise is being mediated by a secondary Ca^2+^ increase through the ryanodine receptors. The initial early rise in cytoplasmic calcium coincides with the phase of spastic paralysis produced by levamisole while the flaccid paralysis of levamisole occurs during the larger, delayed peak.

### The duration of paralysis and recovery, depend on the cholinergic anthelmintic

**Figure 3:**
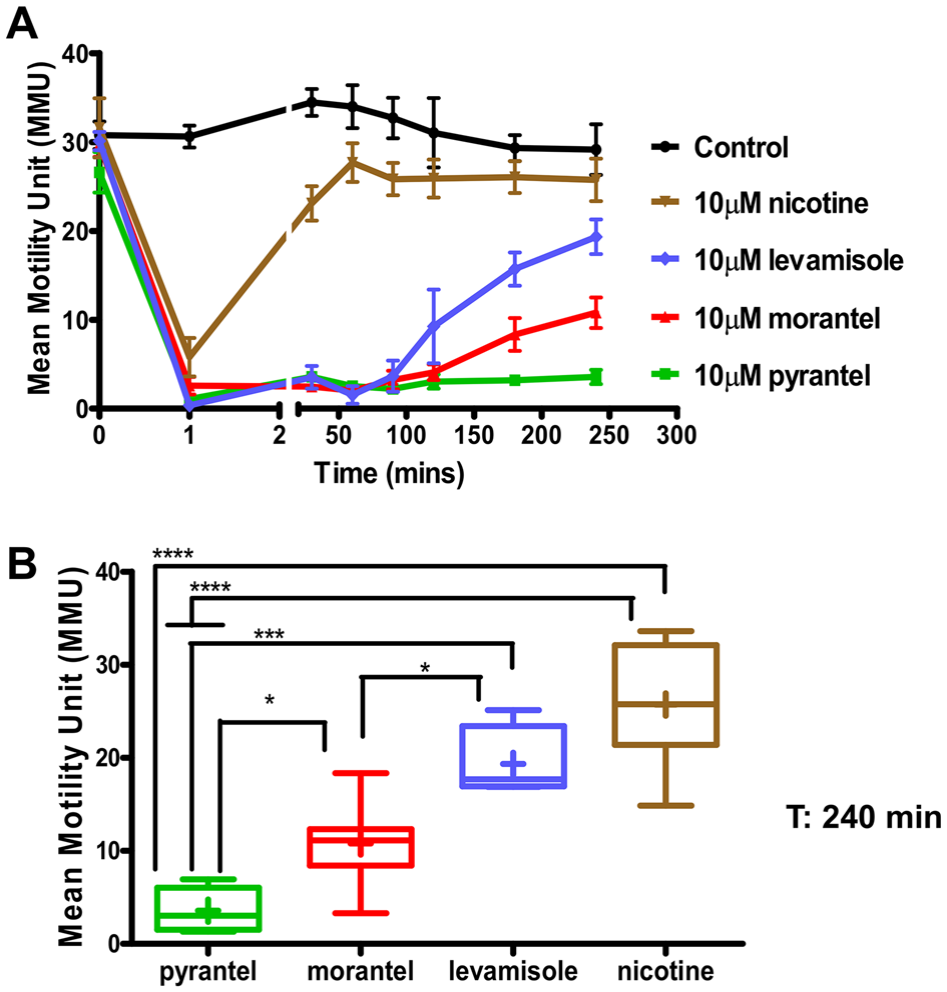
Adult female *B. malayi* possess multiple nAChR subtypes that vary in sensitivity to various nAChR agonists. (**A**) Concentration- and time-dependent analysis of the inhibitory effects of nicotine (brown), levamisole (blue), morantel (red), and pyrantel (green) on worm motility. Worms were treated at a concentration of 10 µM for each drug individually. N = 7 worms per concentration over two biological replicate studies. (**B**) Bar chart (mean ± SEM) showing the mean motility of *B. malayi* after 4 h (240 min) post agonists treatment. The efficacy in inhibition of motility at 240 min was as follows: 10 µM pyrantel > 10 µM morantel > 10 µM levamisole > 10 µM nicotine; n = 7 worms per concentration over two biological replicate studies. One-way ANOVA was performed for statistical analysis and p < 0.05 (*), p < 0.001 (***) and p < 0.0001 (****) were considered statistically significant.

The 4 muscle AChR subtypes, ***L****-, ****P****-, ****N****-* and ***M***-AChRs of *B. malayi* are each preferentially activated by the cholinergic anthelmintics that they are named after: **L**evamisole, **P**yrantel, **N**icotine and **M**orantel (Verma et al. 2017). We tested long duration applications of 10 µM concentrations of levamisole, pyrantel, morantel and nicotine on naïve *B. malayi*, Fig. 3A. All agonists produced a rapid spastic paralysis that inhibited motility by 1 min that was followed by a flaccid paralysis and then a recovery of motility (habituation).

The speed of habituation was anthelmintic-dependent with nicotine showing recovery in less than 50 min, Fig. 3A. The levamisole treated *B. malayi* recovered from the flaccid paralysis, but the recovery was slower than that of nicotine and took 80–240 min. The recovery was even slower for morantel and slower again for pyrantel. Thus, the time required for habituation was anthelmintic-dependent. At 240 min, level of recovery was: nicotine > levamisole > morantel > pyrantel, Fig. 3B. The different rates of recovery of the cholinergic anthelmintics are consistent with these different anthelmintics having selective effects on the different AChR subtypes. Additionally, the lack of sustained paralysis seen over the 4-h period for worms treated with nicotine and levamisole suggest that agonists like pyrantel and morantel, as slow-habituating anthelmintics, could have therapeutic advantages.

### 30 μM levamisole produces desensitization of *L-AChRs* with little or no effect on the other receptor subtypes

**Figure 4:**
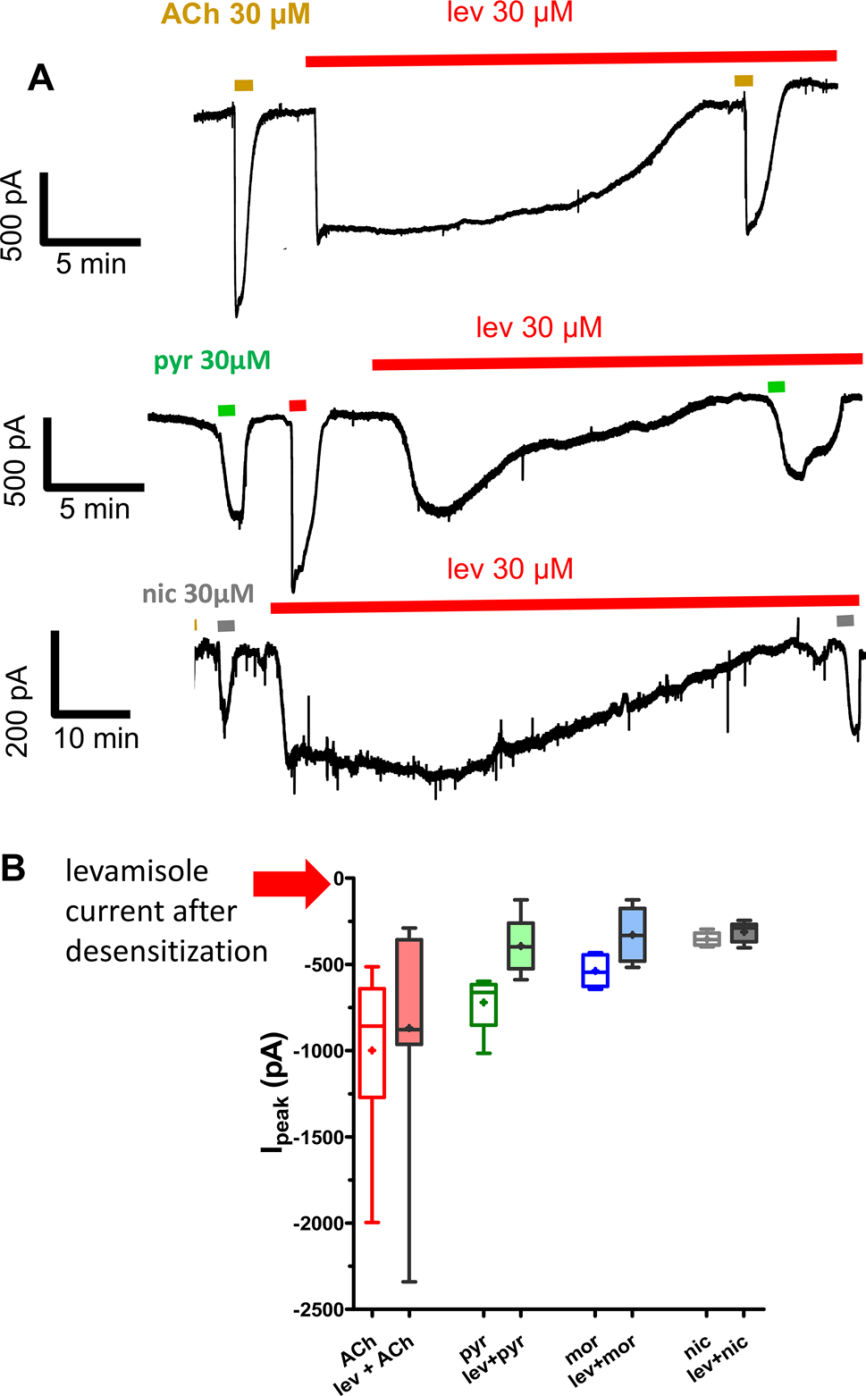
*B. malayi* muscle cells desensitize to continuous levamisole exposure under whole-cell patch-clamp without losing sensitivity to other nicotinic agonists. (**A**) Representative whole-cell patch-clamp recordings demonstrating cells desensitize with loss of inward current, 15–30 min after continuous application of 30 µM levamisole. Inward currents were observed when the same preparation was exposed to nAChR agonists (30 µM ACh, pyrantel, nicotine and morantel, respectively). (**B**) Bar chart demonstrating inward current before and after levamisole desensitization. Note only pyrantel and morantel showed a significant reduction, paired *t-*test, n = 5 from 5 different worms, p < 0.05.

We sought to follow the habituation and adaptation to levamisole by applying a continuous concentration of 30 µM levamisole under whole-cell patch-clamp conditions. Figure 4A shows that the inward levamisole current is not maintained and that it gradually declines over 20–100 min. We tested the response to a 1 min application of 30 µM acetylcholine or a 1 min application of 30 µM anthelmintic before the long application of levamisole. When desensitization was complete, we again measured the responses to the application of acetylcholine or the test anthelmintic. Figure 4B shows a plot of the amplitudes of the cholinergic anthelmintics currents before and after the levamisole desensitization. The levamisole currents reduced to zero (100% reduction) but the reduction in the response to acetylcholine, pyrantel, morantel and nicotine did not reach statistical significance (Fig. 4B, one-way ANOVA, Bonferroni, 5 worms p > 0.05). The desensitization of the levamisole response was much greater than that of the responses to acetylcholine and the other anthelmintics.

### 100 μM levamisole inhibits/desensitizes all receptor subtypes

**Figure 5:**
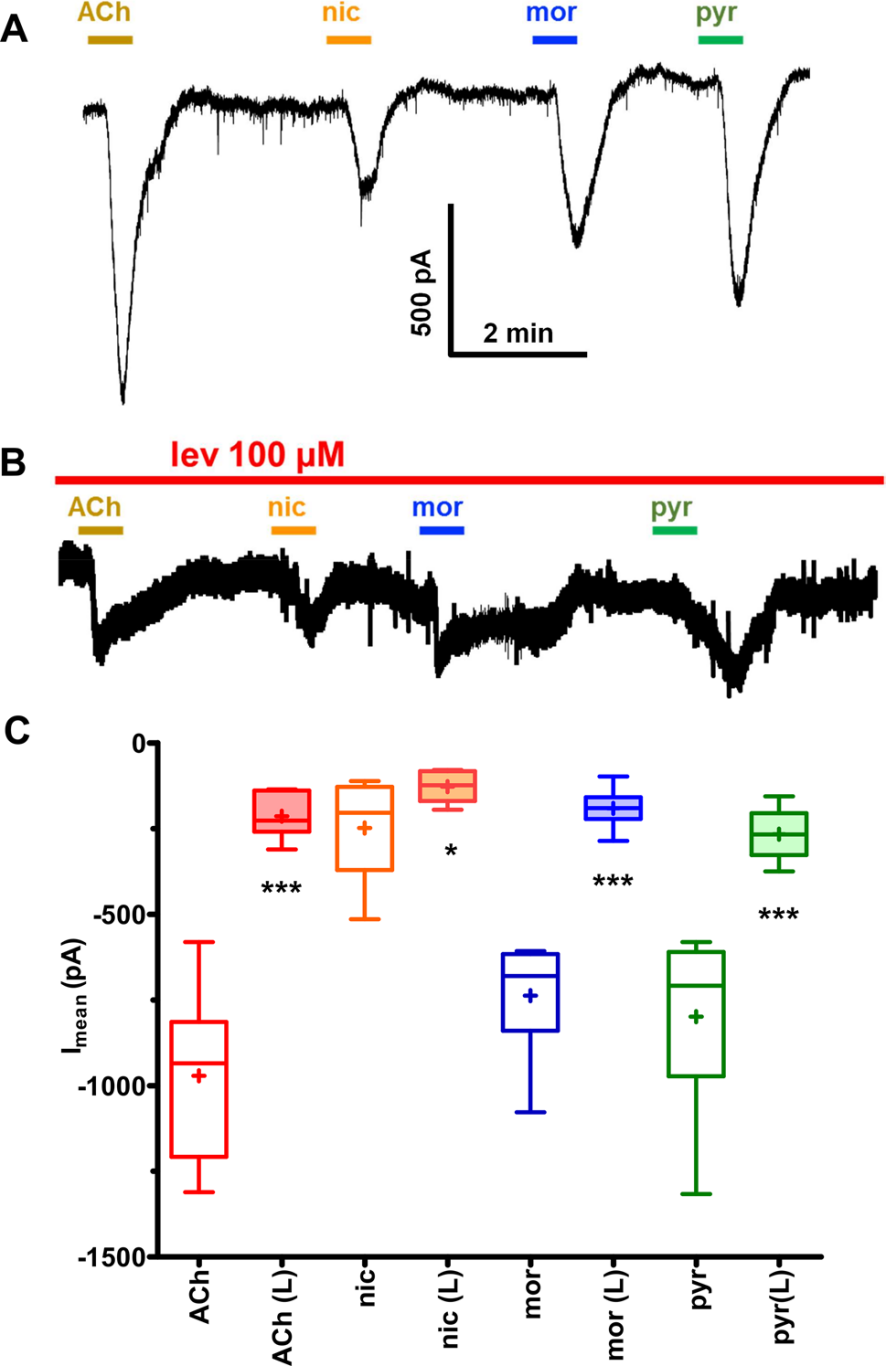
*B. malayi* whole worms after long term whole worm levamisole exposure loses agonist sensitivity in muscles under patch-clamp. (**A**) Representative single electrode patch-clamp recording from *muscle* cells of control *B. malayi* with no prior levamisole exposure demonstrating inward current produced by different nicotinic agonists (ACh, pyrantel, nicotine, and morantel) each at 30 µM concentration for 30 s. (**B**) Representative single electrode patch-clamp recording from *B. malayi* muscle cells after 4 h 100 µM levamisole exposure shows reduction inward currents to different nicotinic agonists (ACh, pyrantel, nicotine and morantel) each at 30 µM concentration for 30 s in the presence of 100 µM levamisole**.** (**C**) Bar chart demonstrating comparative inward currents in muscle cells treated with 30 µM ACh, pyrantel, nicotine and morantel in: control vs the worms maintained in 100 µM levamisole for 4 h (paired *t-*test, n = 6, p < 0.001; p < 0.05 for nicotine).

We then reasoned that a higher concentration of levamisole, 100 µM applied for a longer duration, would have a less selective inhibitory effect on the nAChRs than 30 µM levamisole. We performed electrophysiology on the body wall muscle cells under whole-cell patch-clamp in 5 different recovered *B*. *malayi* female worms after 4 h of long-term 100 µM levamisole exposure. The recordings made in the presence of 100 µM levamisole showed significantly reduced currents for all the AChR agonists tested, Fig. 5. The percent reduction potency series was levamisole (100%) > ACh (78%) > morantel (74%) > pyrantel (66%) > nicotine (48%). The high concentration of levamisole blocked all ***L****-AChR* responses to levamisole but responses to the other agonists of the other AChR subtypes remained, although reduced. These remaining AChR subtypes will be available to contribute to the recovery of motility.

### *unc-38* is upregulated, and *nra-2 *is downregulated, in levamisole habituated *Brugia*

**Figure 6:**
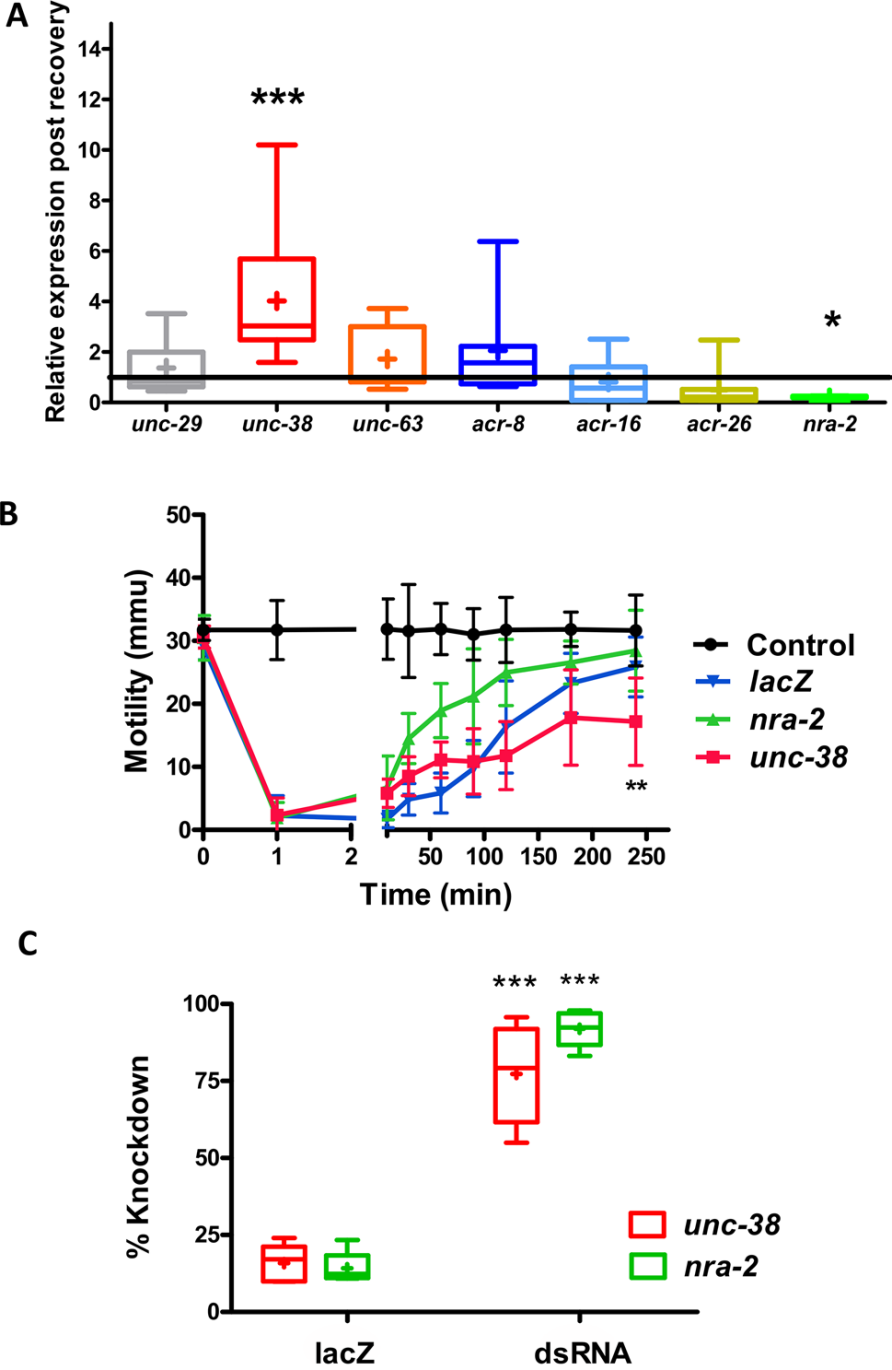
*unc-38* and *nra-2* transcript levels altered during levamisole recovery and *nra-2* knockdown results in faster recovery in adult female *B. malayi.* (**A**) Transcript level analysis on recovered worms after being incubated in levamisole for 4 h. *unc-38* was significantly upregulated while *nra-2* was significantly downregulated (Student’s t-test; ***p < 0.0005, *p < 0.05) whereas the other transcripts tested, *unc-29, unc-63, acr-8, acr-16* and *acr-26* were unaltered. N = 12. (**B**) Knockdown of *nra-2* using dsRNA significantly increases the rate of recovery (Two-way ANOVA; p < 0.05), whereas knockdown of *unc-38* transcript resulted in the worms not completely recovering after 4 h (Student’s t-test; p < 0.005). *lacZ* dsRNA was used as non-specific control and control worms were soaked in water. (**C**) Knockdown of *unc-38* and *nra-2* transcripts after 4 days of incubation in dsRNA assayed using qPCR. Knockdown in the *unc-38* transcript was 77.27 ± 7.21% and *nra-2* was 91.97 ± 2.57%). The knockdown of these transcripts in worms soaked in non-specific *lacZ* control was: *unc-38—*15.87 ± 2.69%; and *nra-2—*14.23 ± 2.32%. N = 5 worms over two biological replicate studies.

We have seen that adult female *B. malayi* treated with 100 µM levamisole adapts, allowing motility to recover after 4 h, although with a slightly modified motility phenotype. We looked for changes in expression levels of the AChR subunits: *unc-38, unc-29, unc-63, acr-8, acr-16* and *acr-26.* We hypothesized that expression of one or more of these subunits would change and be involved in the habituation. We point out that the *B. malayi* genome contains two alternatively spliced copies of *unc-38* and *unc-63;* to measure expression we made primers at exons common to both their splice variants*.* We observed that in the recovered worm, the *unc-38* transcript was upregulated, while the expression of the other genes was unaffected. We also observed that *nra-2* (a gene that encodes for an ER retention protein) was downregulated, Fig. 6A. *nra-2* in *C. elegans* that leads to faster adaptation to levamisole^19,20^. These observations indicate a role for *nra-2* and *unc-38* in part of the habituation response to levamisole.

### *nra-2* knockdown speeds but *unc-38* knockdown slows levamisole desensitization/recovery

To determine the effect of *nra-2* on the adaptive response to levamisole*,* we knocked down the *nra-2* transcript by soaking the worms with dsRNA for four days. We then found that these worms recovered faster than control *lacZ* worms in 100 µM levamisole, Fig. 6B. qPCR on these worms confirmed knockdown of the *nra-2* transcript, Fig. 6C. We also knocked down *unc-38* transcript and found that these worms recovered more slowly from levamisole and recovery took longer, Fig. 6B. The RNAi experiments and expression changes of *unc-38* and *nra-2* indicate that these two genes are key components of the habituation response of *B. malayi* to levamisole.

### *nra-2* knockdown reduces levamisole currents but not ACh currents

**Figure 7:**
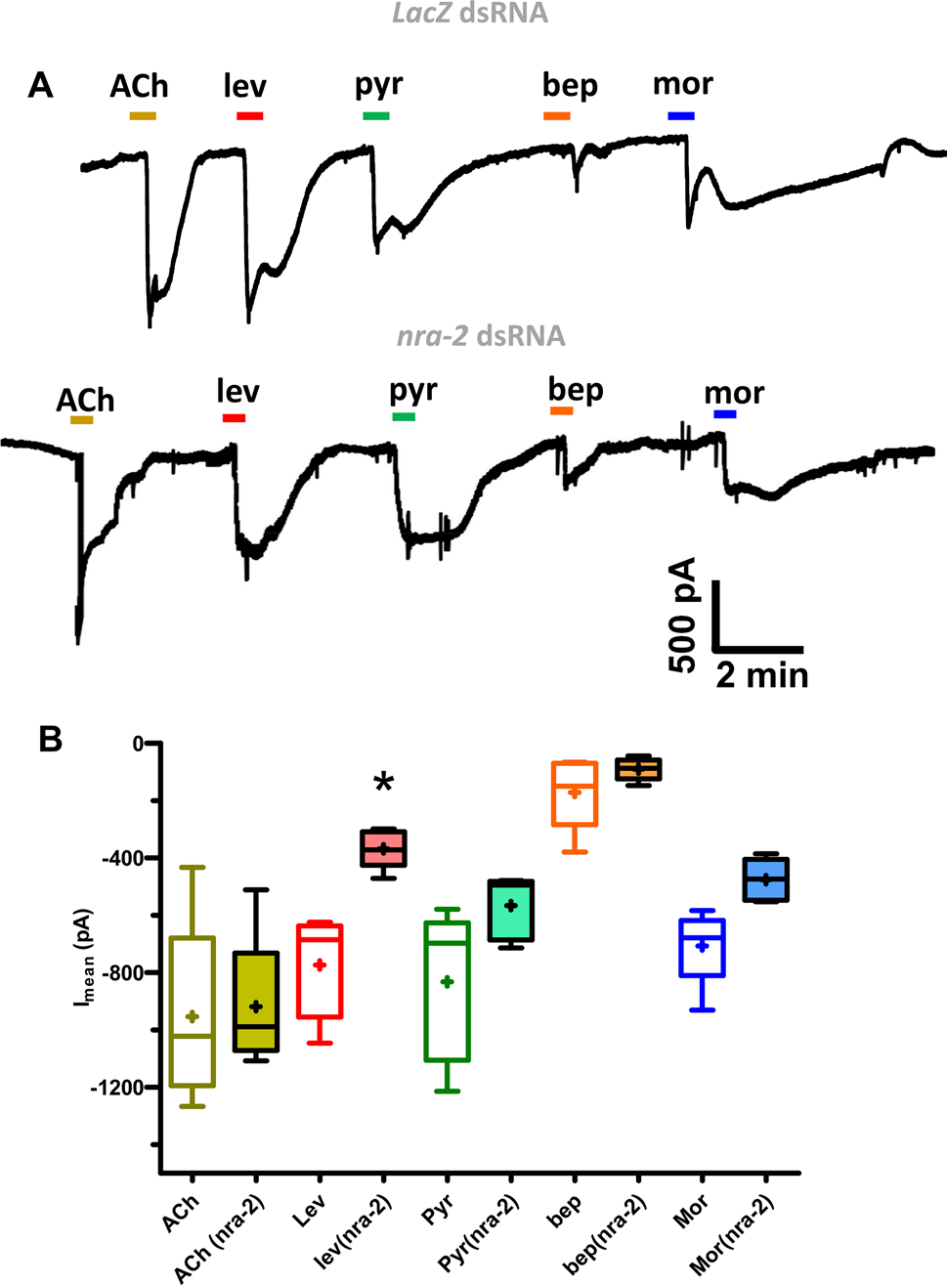
Whole-cell patch-clamp recording from muscle cells of the *nra-2* dsRNA treated adult worms. (**A**) Representative whole-cell patch-clamp recordings demonstrating that adult worms soaked in *LacZ* dsRNA treated worms produced inward currents (30 µM ACh, pyrantel, bephenium and morantel, respectively) and the inward current response to levamisole and pyrantel were significantly reduced in *nra-2* dsRNA treated worms. (**B**) Histogram demonstrating inward current response to (30 µM ACh, pyrantel, bephenium, and morantel) in *LacZ* and *nra-2* dsRNA treated worms. The responses to levamisole and pyrantel were significantly reduced **(**paired *t-*test, p < 0.005, n = 5 cells from 5 worms).

To examine the effects of *nra-2* further, we made whole cell patch-clamp recordings from muscle cells treated with dsRNA for 4 days to knockdown *nra-2*, Fig. 7. The muscle cells were each given a standard perfusion for 25 s with 30 µM concentrations of acetylcholine, and levamisole. We focused on levamisole and acetylcholine responses and for controls also tested 25 s with 30 µM concentrations of pyrantel, morantel and bephenium. We measured the mean peak currents measured in 5 separate preparations in *LacZ* treated control worms and dsRNA *nra-2* treated worms*.* There was a selective reduction in the amplitude of the levamisole currents that was statistically significant but not with acetylcholine or the controls, Fig. 7B. Again, we see evidence of agonist selective effects of *nra-2* knockdown on the response to levamisole. The reduction in the levamisole sensitivity on the muscle shows that *nra-2* is required for maintaining and fostering the ***L****-AChRs.*

### Reversal of levamisole habituation and *nra-2* expression levels after removing levamisole

**Figure 8:**
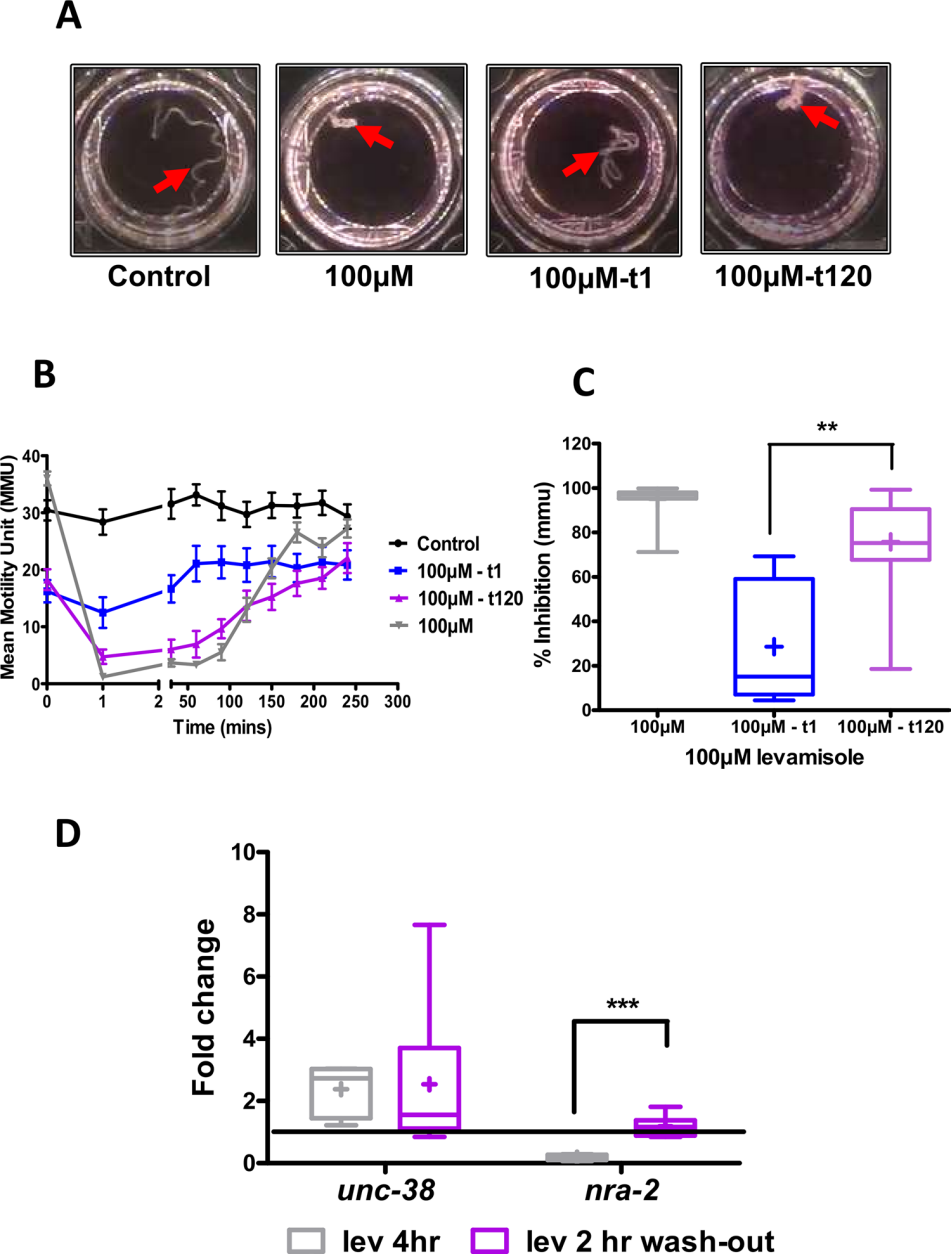
Treated adult female *B. malayi* become re-sensitive to levamisole after 120 min in RPMI media. (**A**) Shows images of worms under spastic paralysis for t-0, t-1 and t-120 worms. (**B**) Worms treated in levamisole recover after 4 h (t-0; grey). These worms were transferred into RPMI media and treated with a second dose of levamisole after 1 min (t-1; blue) and 120 min (t-120; purple). Control worms (black) were treated with water. (**C**) Histogram of the spastic paralysis effect of levamisole on the above worms. Inhibition of motility in t-1 worms were significantly lower compared to the t-120 worms (Student’s t-test; p < 0.005; n = 8 over two biological replicates). (**D**) Transcript levels of *nra-2* in worms recovered under levamisole after the first 4 h (t-0 worms) and in worms moved to fresh RPMI media for 120 min (t-120). N = 6 over two biological replicates.

We looked to see if there was a reversal of habituation after removal of levamisole. We treated *B. malayi* with 100 µM levamisole for 4 h as before. We then transferred these worms to fresh media without levamisole and then tested groups of 4 worms with 100 µM levamisole after an interval of 1, 30, 60, 90 or 120 min, Fig. 8A,B. The worms re-tested with levamisole 1 min after habituation in the fresh media (t1: 1 min) showed little response to levamisole with only a 27.8 ± 15.8%, reduction in motility, Fig. 8B,C. The worms tested with levamisole after 120 min (t120) in fresh media responded well to the second exposure to levamisole with a 70.65 ± 7.59% reduction in motility, Fig. 8B,C. Figure 8C shows that the inhibition of motility with the t-1 worms was significantly smaller than the t-120 worms.

We also investigated the change of expression of *nra-*2 and *unc-38* following return to fresh levamisole-free media, to determine if there were expression changes associated with the loss of this habituation. The transcript levels were measured in: (i) naïve untreated worms; (ii) in control worms habituated after 4 h in levamisole and; (iii) in worms habituated for 4 h and then moved to fresh RPMI media for 120 min. The expression level of *nra-2* was decreased and *unc-38,* was increased in habituated worms, Fig. 8D, as before (see Fig. 6A). However, the *nra-2* transcript level of worms removed from levamisole and soaked in fresh media for 2 h (4 h + 2 h) returned to the baseline level, but the *unc-38* expression appeared to be unchanged at this time, Fig. 8D. Here, we see again the correlation between the response to levamisole and expression of *nra-2*.

### Recovery and knockdown of AChR receptor genes

**Figure 9:**
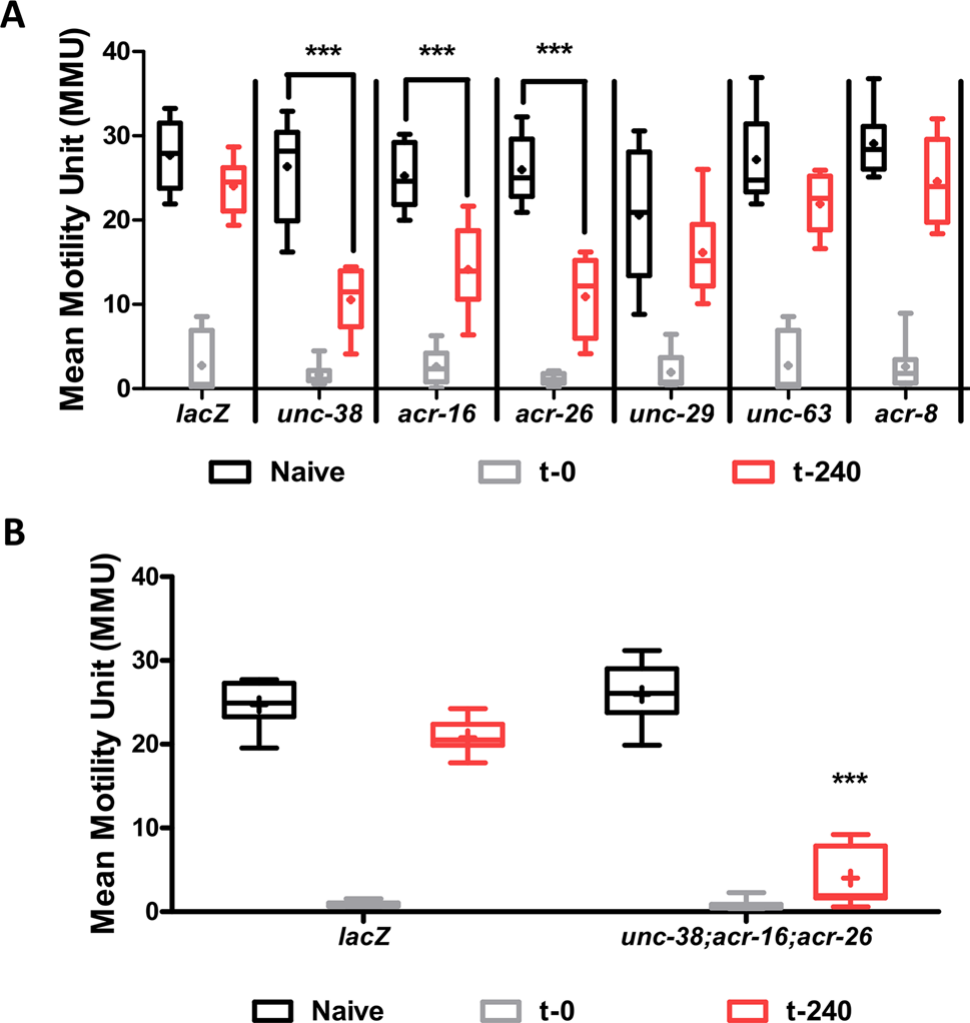
UNC-38, ACR-26 and ACR-16 are required for recovery of motility with 100 µM levamisole treatment (habituation). (**A**) Shows the effect of knockdown on *unc-38*, *unc-63, unc-29, acr-8, acr-16* and *acr-26* on levamisole habituation. Knockdown of *unc-38, acr-16* and *acr-26* caused significant impairment in levamisole habituation at t-240 min. (**B**) Triple knockdown of *unc-38, acr-26* and *acr-16* resulted in no reduction in motility in naïve worms, but the levamisole habituation phenotype was severely impaired (2-way ANOVA, p < 0.05, n = 8 over two biological replicates).

Figure 9A shows that the knockdown of single nAChR subunit genes (*unc-38, unc-63, acr-8, acr-26* and *acr-16*) had little detectable effect of the spontaneous motility of *B. malayi*. Knockdown of *unc-29* significantly reduced but did not abolish spontaneous motility of worms. This was expected as UNC-29 and UNC-38 together were shown to be necessary for spontaneous motility (Verma et al. 2017): UNC-29 is the only non-alpha subunit in body muscle, so the effect of knocking it down was anticipated to have a bigger impact on motility than knocking down *unc-38,* one of five alpha subunits. Regardless, all these worms showed sensitivity to levamisole and were paralyzed upon treatment with 100 µM levamisole (Fig. 9A).

When we looked at recovery however, we found that knockdown of *unc-38, acr-26,* and *acr-16* delayed recovery, and *unc-63, unc-29* and *acr-8* did not, Fig. 9A. The knockdown of *unc-38, acr-26* and *acr-16* together had a bigger effect and prevented (> 12 h) rather than just delayed recovery, Fig. 9B. Thus, the genes that play a role in levamisole recovery (*unc-38, acr-26,* and *acr-16*) are not essential for normal motility (*unc-29*). There is a change from the genes driving normal spontaneous motility to the genes that that are driving the motility seen with recovery (habituation) to levamisole. This change is associated with the modified motility phenotype, Fig. 1E.

## Discussion

### Dynamic and plastic AChRs in Brugia muscle

Here we have documented more complex time-dependent and concentration-dependent responses of a parasitic nematode to an anthelmintic that is detectable at µM concentrations, Fig. 1A. Plasma concentrations after a 150 mg dose of levamisole in humans exceed these concentrations^31^ indicating that the time-dependent and concentration-dependent effects should occur during therapeutic use. Our observations illustrate some of the limitations of simple anthelmintic concentration–response plots when evaluating efficacy of these drugs. The anthelmintic we studied was levamisole, which is a selective agonist of specific subtypes of nematode nicotinic AChRs in *Brugia malayi.* In *Ascaris suum* and *Nippostrongylus braziliensis,* levamisole also produces time-dependent contractions that pass off with time^32^ as they do in the *C. elegans* model nematode^12^.

**Figure 10:**
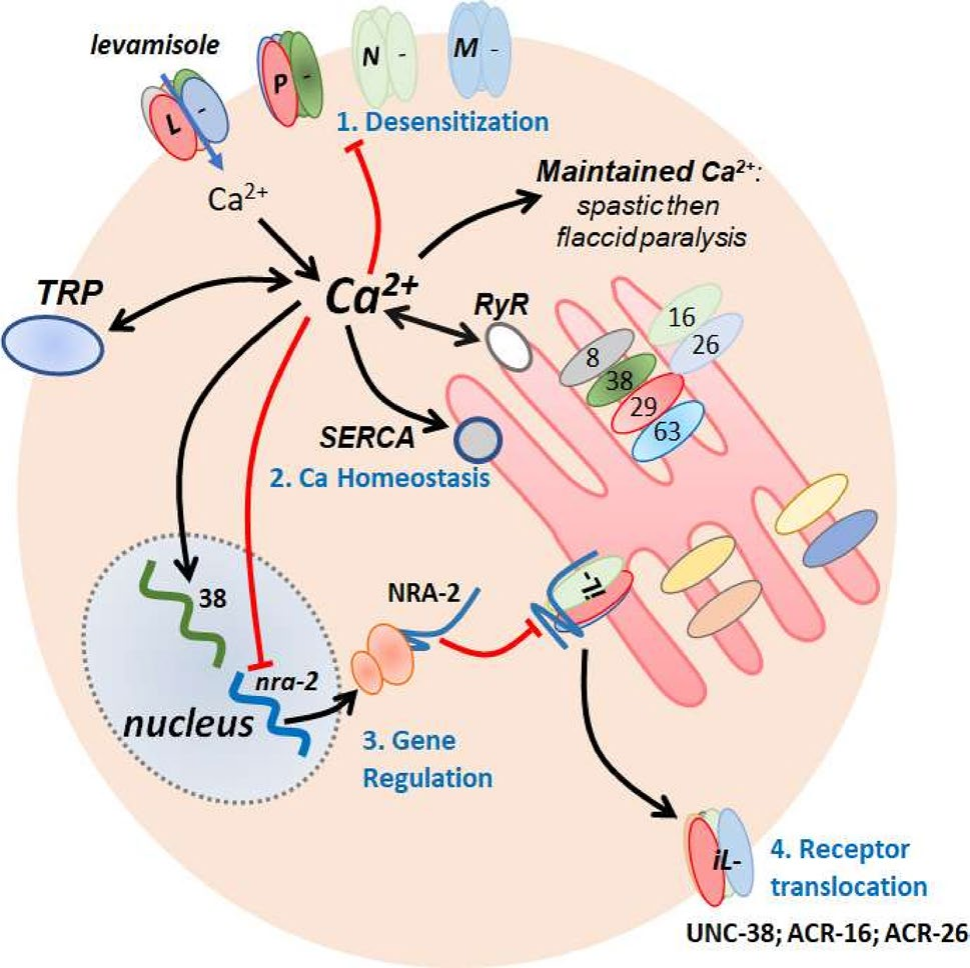
Summary diagram of proposed mechanisms of homeostatic plasticity allowing levamisole habituation. Levamisole preferentially opens the ***L***-subtype of AChRs, to produce depolarization and entry of calcium. The calcium influx in the muscle produces contraction and spastic paralysis. The ***L***-subtype of AChRs desensitizes over ~ 20 min (**1:** Desensitization). Calcium also builds up in the cell with the support of the calcium-induced calcium release via the ryanodine receptor (RyR). The sarcoplasmic calcium then gradually decreases due to homeostatic uptake mechanisms (**2:** Ca Homeostasis) that include the sarcoplasmic endoplasmic reticulum calcium ATPase (SERCA). *unc-38* is upregulated while *nra-2* is down regulated (**3:** Gene Regulation) that leads to a dis-inhibition of insensitive levamisole AChRs (***iL-***) in the ER containing UNC-38, ACR-26 and ACR-16 (**4:** Receptor translocation). The ***iLs*** are then translocated to the membrane facilitation the recovery of motility of the worms.

In *C. elegans* there are two nicotinic AChR subtypes present on muscle: the nicotine-sensitive ***N-***AChR and the levamisole-sensitive ***L-***AChR^22^. In *Ascaris suum* there are three separable AChRs: ***L****-, ****N-****,* and ***B-***subtypes^33^. In *Brugia malayi* muscle, Fig. 10, there are four separable subtypes: ***L****-, ****P****-, ****M****-* and ***N****-*subtypes^17^. Levamisole preferentially activates ***L****-*AChRs; pyrantel preferentially activates ***P-***AChRs; morantel preferentially activates ***M-***AChRs; bephenium preferentially activates ***B-***AChRs and; nicotine preferentially activates ***N-***AChRs. Parasite muscle AChR subtypes are produced by different pentameric combinations of nAChR subunits that include UNC-38, UNC-29, UNC-63, ACR-8, ACR-16 and ACR-26. The AChR subunits combine in different pentameric and stoichiometric arrangements to produce the AChR subtypes that have distinctive pharmacological properties and sensitivities to levamisole^24,28,34,35^.

The numbers of the different AChR subtypes will alter whole muscle current responses and muscle contraction responses to different cholinergic anthelmintics. *B. malayi* muscle responses are characterized by an initial spastic paralysis, followed by flaccid paralysis and then a gradual recovery of motility over 4 h. We see desensitization (decline) of the levamisole current response when 30 µM levamisole is applied for a long time (~ 20 min) while current responses to acetylcholine remain are not desensitized. Long term (4 h) application of levamisole increases *unc-38* and decreases *nra-2* expression; *nra-2* knockdown also selectively reduces levamisole current responses, Fig. 7B. In *C. elegans* AChRs sensitive to levamisole are also decreased and AChRs insensitive to levamisole are increased in *nra-2* null mutants^19^. These observations reveal dynamic mechanisms of AChRs in parasitic nematodes by which responses to levamisole are modified allowing adaptation to limit effects of levamisole.

What is the functional significance of the plastic and different nAChR subtypes on muscle cells? The nAChR subtypes are all non-selective acetylcholine-gated ion-channels that differ in their calcium permeability, sensitivity to acetylcholine, rate of desensitization and perhaps membrane location. The levamisole-preferring, ***L***-AChR, is made up of the subunits of UNC-38, UNC-63, ACR-8, and UNC-29 and is 20× more permeable to calcium than channels composed of UNC-63, ACR-8 and UNC-29 subunits^28^. The AChR channel that desensitizes faster than others, is the ***N****-*AChR, that is composed of ACR-16 subunits^36^ and that is selectively activated by nicotine and acetylcholine rather than levamisole, pyrantel and morantel. The effects of nicotine on motility desensitizes faster than levamisole which in turn desensitizes faster than morantel and pyrantel, Fig. 3A.

Figure 10 illustrates a summary diagram with the different subtypes of nAChRs found on *B. malayi* muscle membrane: it shows the editing of the AChR subtypes by NRA-2 inhibiting the insertion of levamisole insensitive AChRs into the plasma membrane. Figure 10 also illustrates how the increased cytosolic calcium that we have observed with prolonged levamisole application may promote *unc-38* expression, inhibit *nra-2* expression, inhibit motility, interact with TRP channels^37^ and contribute to AChR desensitization.

The general processes of homeostatic plasticity are well recognized in neuroscience and refer to the capacity of excitable cells to regulate their own activity. Intracellular calcium is the primary coordinator that regulates their excitability with compensatory adjustments occurring over different time scales. The plasticity involves changes in the number and distribution of membrane ligand-gated ion-channels, voltage-activated ion-channels, calmodulin, calcineurin, intracellular calcium release and uptake^10,38,39^. In *C. elegans* the plasticity is associated with regulation of the number of AChRs presented to the plasma membrane^40^ and with AChR subunit composition and transfer to the plasma membrane being regulated by NRA-2^19^*.* The homeostatic plasticity will allow the parasite to control and adapt motility to different environments that the nematode finds itself in as it moves through its life cycle and around in the host. In some environments, it will need to more rapid contractions for swimming, in others it will need a slower sustained contraction to hold onto its location. It will also allow the parasite to adapt to the cholinergic anthelmintics as we have seen.

### Desensitization and flaccid paralysis

The effects of levamisole on motility are time- and concentration-dependent and characterized by the initial spastic paralysis phase (< 20 min) due to the opening of nAChR channels on body muscle; this is followed by a phase of flaccid paralysis as the body muscles relax (from the end of spastic paralysis to < 4 h), despite elevated cytosolic calcium levels, and AChR channel desensitization. The features of mammalian nicotinic receptor channel desensitization have been studied extensively^41–43^ and involve different mechanisms including slow adjustments of the channel amino-acid positions and changes is receptor phosphorylation driven by calcium dependent kinases and phosphatases. The regulators of desensitization of AChRs that are present in *C. elegans* muscle and *Brugia* include: TAX-6, a calcineurin A subunit, that affect desensitization of AChRs in rat chromaffin cells^44^; SOC-1 (a multi-subunit adaptor protein); and PLK-2 (a serine/threonine kinase^44,45^. We have not addressed these regulators in this study, but they are anticipated to affect the rate of levamisole desensitization and homeostatic plasticity.

### Calcium homeostasis

Maintained application of levamisole, Fig. 2, produces an initial modest increase in cytosolic calcium followed by secondary larger increase in cytosolic calcium, Fig. 10. Ryanodine receptors (RyRs: UNC-68) are present in nematode parasite muscles (*Ascaris*^30,46^; *Brugia*: Wormbase) as well as *C. elegans,* UNC-68: Wormbase^47,48^. The rise in cytosolic calcium following opening of AChRs by levamisole then produces muscle contraction mediated by the calcium contraction coupling pathway^46,49,50^. TRP channels (TRP-2, GON-2 and CED-11) channels present in *Brugia* muscle also contribute to the muscle contraction^16,51,52^. TRP channels are subject to regulation by intracellular messengers including direct or indirect activation and inhibition by calcium and PKC^51,53,54^, Fig. 10. Inhibition of the TRPs will contribute to calcium homeostasis as will calcium uptake: (i) ER and plasma membrane calcium ATPase (SERCA: *sca-1a* & *sca-1b*:^55^; (ii) Na/Ca exchangers; (iii) Na/Ca/K exchangers; and (iv) Ca/cation exchangers, *ncx-1 to ncx-10*^56^.

Our calcium fluorescence experiments, Fig. 2, indicate that cytosolic calcium remains elevated during the earlier part of the flaccid paralysis indicating that that calcium homeostasis is not the explanation for the flaccid paralysis. One explanation that allows a relaxation despite elevated calcium is a calcium-induced calcium-desensitization of the myosin light chain by dephosphorylation with myosin light chain phosphatase^57–59^. Figure 5 shows that application of 100 µM levamisole for 4 h blocks the levamisole currents and inhibits but does not block currents from of other AChRs. These remaining AChRs, once the high calcium concentration of sarcoplasm has been cleared by the homeostatic mechanisms^60,61^ could permit the return of motility as the sensitivity of myosin light chain kinase returns as the excess cytoplasmic calcium is removed. Inhibition of the homeostatic mechanisms is anticipated to inhibit the return of motility.

### Gene regulation and recovery

We tested for the recovery from levamisole desensitization following removal of the levamisole, Fig. 8. We find that there was a gradual return of sensitivity over 4 h associated with a return towards control *nra-2* message levels but with *unc-38* levels remaining elevated. NRA-2 is expressed in endoplasmic reticulum of body wall muscle in *C. elegans* where it controls the composition of different AChR subunits in the plasma membrane so that *nra-2* null mutants are less sensitive to levamisole. NRA-2 is a homolog of human nicalin that contains a predicted calcium-sensitive EF hand that extends into the lumen of the endoplasmic reticulum suggesting actions related to emptying of calcium from the ER.

### Therapeutic significance

We have seen that *B. malayi* can adapt to the anthelmintic levamisole by desensitization of the levamisole sensitive receptors (***L****-*AChRs) on muscles. During treatment of an infected host with levamisole there will be gradual rather than a rapid rise in the concentration of levamisole at the site of the worm. This time-dependent increase in levamisole concentration at the site of the worm can permit adaptation and homeostatic plasticity processes that we have described to allow survival and resistance to treatment.

The desensitization and homeostatic plasticity mechanisms that we have observed are driven by changes by increases in intracellular calcium and could also be at the heart of the transient effect of diethylcarbamazine^16^. For these drugs to be effective particularly against nematode parasites that are less sensitive to drugs like levamisole and diethylcarbamazine, then repeated dosing allowing some recovery from the habituation may enhance a therapeutic benefit^62^.

## Conclusion

We have seen that the response to an anthelmintic levamisole is time dependent as the parasite adapts to the presence of the drug. There is a spastic paralysis, a flaccid paralysis, and then recovery and change in nAChR subtypes present on the muscle cells. It is proposed that the nAChR subtypes are dynamic, showing a homeostatic plasticity and that activity and cholinergic anthelmintics drive the subunit composition and thereby change the nAChR subtype. Such a mechanism will allow the nematode parasite to accommodate to an environmental change as well as exposure to an anthelmintic. Homeostatic plasticity is an unappreciated mechanism for anthelmintic resistance.
